# Adsorption of a water-soluble molecular rotor fluorescent probe on hydrophobic surfaces

**DOI:** 10.1038/s41598-022-26722-w

**Published:** 2022-12-23

**Authors:** Elham Mirzahossein, Marion Grzelka, Fabrice Guerton, Daniel Bonn, Ross Brown

**Affiliations:** 1 Van der Waals-Zeeman Institute, Institute of Physics, University of Amsterdam, 1098XH Amsterdam, The Netherlands; 2 Université de Pau et des Pays de l’Adour, E2S UPPA, CNRS, IPRA, Pau, France; 3 Université de Pau et des Pays de l’Adour, E2S UPPA, CNRS, IPREM, Pau, France

**Keywords:** Surfaces, interfaces and thin films, Confocal microscopy

## Abstract

Environmentally sensitive molecular rotors are widely used to probe the local molecular environment in e.g. polymer solutions, polymer glasses, and biological systems. These applications make it important to understand its fluorescence properties in the vicinity of a solid surface, since fluorescence microscopy generically employs cover slides, and measurements are often done in its immediate vicinity. Here, we use a confocal microscope to investigate the fluorescence of (4-daspi) in glycerol/water solutions close to the interface using hydrophilic or hydrophobic cover slips. Despite the dye’s high solubility in water, the observed lengthening of the fluorescence lifetime close to the hydrophobic surface, implies a surprising affinity of the dye with the surface. Because the homogeneous solution and the refractive index mismatch reduces the optical sectioning power of the microscope, we quantify the affinity with the help of a simple model of the signal *vs.* depth of focus, exhibiting surface and bulk contributions. The model reduces artefacts due to refractive index mismatch, as supported by Monte Carlo ray tracing simulations.

## Introduction

Fluorescence of organic molecules arises from transitions between electronic states with high peripheral densities, making it exquisitely sensitive to perturbation by the molecular environment at nanometre scales. This sensitivity underpins numerous analytical applications, in which fluorescence spectroscopy, both wavelength- and time-resolved, informs e.g. on the polarity, the pH, the viscosity of the medium^1,2^, or the local elastic stress in solids^3–5^. The challenge for aqueous applications is striking a balance between conjugation to ensure strong absorption and emission in the visible to near IR spectral range, and electron localisation due to peripheral substitutions with more water-soluble polar groups (perylene di-imide for example). Indeed, many prominent water-soluble dyes are salts, e.g. the rhodamines.

An interesting fundamental question is then the equilibrium between free-swimming species in aqueous solution and molecules adsorbed to an immersed substrate, and the influence of the substrate’s surface state. Furthermore, practical consequences may arise in popular and even smaller microfluidic devices. For example, if a solution of fluorophore is at equilibrium with the adsorbed species at respective bulk and surface number densities $\rho$ and $\sigma$, in a tube of diameter 2*r*, the ratio of adsorbed to bulk molecules is $N_{\hbox {ads}}/N_{\hbox {bulk}}=2\sigma /(r\rho )$. Counting a molecular surface area $s_{\hbox {mol}}\approx 1$ nm$^2$ and concentration $\rho \approx 10^{-6}$ M, typical for applications, $N_{\hbox {ads}}/N_{\hbox {bulk}}$ would be $\approx 0.3$ at 1% monolayer coverage in a 200 $\upmu$m diameter tube, *i.e.* $\sigma \approx 10^{-2}$ nm$^{-2}$. The mean surface density of free, or fully isolated silanols on silicas^6^, that could most readily adsorb a dye molecule, is larger, of the order of 0.1 nm$^{-2}$, as deduced from non-linear and evanescent wave cavity ring-down spectroscopies^7,8^. Thus, adsorption of fluorescence dyes might be non negligible and induce distortion of bulk fluorescence.

The molecular rotors are a class of dyes particularly sensitive to their environment, useful for probing interfaces and viscosity. Upon photoexcitation in free solution, they tend to form twisted intramolecular charge transfer states (TICT) with rapid non-radiative decay *via* conformational relaxation^9^. But when the local environment hinders such relaxation, e.g. by viscosity or proximity of an interface, twisting is impeded and the fluorescence increases. Therefore, the fluorescence quantum yield of molecular rotors strongly depends on the nature of the direct environment of the dye such as microviscosity and polarity^9–13^.

**Figure 1:**
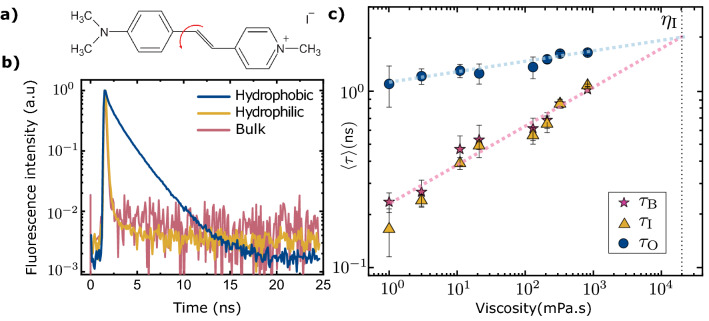
Fluorescence decays of 4-daspi reveal an adsorbed layer on hydrophobic glass Fluorescence of 4-daspi in glycerol/water solutions. (**a**) Structure of the molecular rotor 4-daspi. The red arrow indicates the intramolecular rotation; (**b**) Decays in water in the bulk (red) and at a hydrophilic interface (yellow) are identical. The longer decay at a hydrophobic interface (blue) shows up an additional, physisorbed species; (**c**) Amplitude averaged lifetime of 4-daspi as a function of glycerol/water viscosity. Hydrophobic glass: $\tau _{\textrm{O}}$, bullets; hydrophilic glass: $\tau _{\textrm{I}}$, triangles; bulk for the reference measurements: $\tau _{\textrm{B}}$, stars. Error bars indicated the standard deviation for different samples. The dashed lines are Förster-Hoffman equation fitted to data points of bulk solutions and hydrophobic surface.

In addition to viscosity and polarity, the hydrophobicity (or hydrophilicity) of an interface^14^, temperature and pH of the solvent *etc.* may influence the fluorescence of molecular rotors^10,15,16,13,14,17^. Trans-4-[4-(dimethylamino)styryl]-1-methylpyridinium iodide (4-daspi) (Fig. 1a) is a particularly useful water soluble, cationic molecular rotor^18,19^. Non-radiative decay occurs mainly through rotation around the single bond^20^ indicated in Fig. 1a. The photophysical properties of hemicyanines like 4-daspi have been extensively investigated^20–22^ and widely used for probing bulk properties such as studying water-containing reverse micelles properties^11^, the mechanical properties of viscoelastic media^23^, polymer solutions^24^ and probing red blood cell stiffness^25^. However, their behaviour close to the interface is less clear.

While single molecule methods^26^, specially total internal reflection^27,28,26^ are now a method of choice for optical studies of adsorption of strong fluorophores, low fluorescence quantum yield or low total emitted photons before photobleaching^29^ hamper application of such methods to many molecular rotors.

Here therefore, we show that simple confocal fluorescence microscopy of bulk solutions in equilibrium with a substrate, can provide insight into the adsorption of 4-daspi on glass. We use fluorescence lifetime measurements to distinguish the physi-sorbed and free species of 4-daspi in glycerol/water solution close to hydrophobic and hydrophilic surfaces. Of course, partial loss of optical sectioning power in presence of the bulk, refractive index-mismatched solution leads to an apparent interaction with the interface over several microns. We show how to largely correct this artefact with a simple model involving all-experimental inputs and supported by Monte Carlo ray tracing. No physisorption could be detected on hydrophilic glass, at any glycerol concentration. Whereas one would expect at first that adsorption of the cationic dye to the hydrophobic glass would be less important in pure water than in the mixed solvents, the reverse is the case- significantly, about ten times less adsorption in the more concentrated glycerol solutions.

## Results and discussion

### Lengthening of the fluorescence lifetime of 4-daspi at a hydrophobic surface

A droplet of glycerol/water mixture with variable concentration of glycerol is sandwiched between a hydrophobic and a hydrophilic cover-slip on an inverted confocal microscope and the fluorescence lifetime is determined as a function of the depth of the nominal focus ($z_{\hbox {F}}$) into the solution. As expected, the fluorescence lifetime of 4-daspi deep in the bulk ($\tau _{\textrm{B}}$) increases with increasing viscosity of the solution following the Förster-Hoffman relation^30^. Either the hydrophilic (I) or the hydrophobic (O) interface may be presented towards the objective.

The fluorescence decays of 4-daspi in pure water close to the hydrophobic or hydrophilic surface are compared in Fig. 1b. The average lifetime of 4-daspi in pure water at the hydrophilic surface is $0.12 \pm 0.02$ ns, above the value in water reported in the literature, around 10 ps^11^. This apparent long lifetime must be due to the instrument response of our setup. Importantly, the fluorescence lifetime measured on the hydrophilic surface, $\tau _{\textrm{I}}$, is indistinguishable from that in the bulk and follows the same trend with the viscosity of the solution (Fig. 1b,c). But on the hydrophobic surface, on the contrary, the fluorescence properties differ from the bulk. The lifetime measured at the hydrophobic interface is significantly longer, up to $\tau _{\textrm{O}}=1.7 \pm 0.1$ ns at the interface (99 wt.% glycerol), suggesting that dye is present in different physical states. Such a change might suggest aggregation of the dye as probed in ref^31,32^. However, as the measured lifetime is relatively insensitive to the viscosity of the overlying solvent (Fig. 1c), the dye is not inside the solution . It therefore reveals an adsorbed species. Adsorption was confirmed by depositing a droplet of aqueous 4-DASPI solution on a coverslip, measuring the lifetime, thoroughly rinsing the coverslip and replacing the solution by pure water and finally measuring the lifetime again. Before and after rinsing we find the same, long fluorescence lifetime close to the glass (1.4 ns).

**Figure 2:**
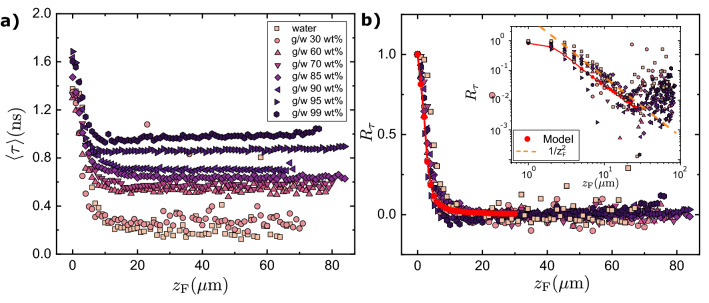
Data collapse of the fluorescence lifetime *vs.* depth of focus Fluorescence lifetime *vs.* nominal depth of focus into the solution (*i.e.* as shown by the microscope stand), $z_{\hbox {F}}$, from the hydrophobic glass at $z_{\hbox {F}}=0$. (**a**) Amplitude averaged lifetime as a function of $z_{\hbox {F}}$ for various glycerol/water solutions; (**b**) Empirical data collapse $R_{\tau }(z_{\hbox {F}})=(\langle \tau \rangle (z_{\hbox {F}})-\tau _{\hbox {B}})/(\langle \tau \rangle (0)-\tau _{\hbox {B}})$. Red curve: normalized distribution function $R_{\hbox {th}}$, Eq. (10), computed by ray tracing simulation from the basic parameters of the microscope and the refractive index of the samples. Inset: Same data, showing the $1/z_{\hbox {F}}^2$ decay of the interface contribution.

Figure 2a shows the dependence of the lifetime on depth of focus close to a hydrophobic interface ($z_{\hbox {F}}=0$). The apparent range of influence of the interface, up to $z_{\hbox {F}}\approx 20$ $\upmu$m, is shown below to be an artefact due to loss of optical sectioning power: The drastic spatial filtering applied by the confocal pinhole to a collection of out of focus point emitters, is strongly curtailed when confocal imaging is applied to 2-D, or worse still 3-D objects, like the present adsorbed layer and the homogeneous solution.

Figure 2b nonetheless shows an empirical but striking data collapse with the range of shortest to longest experimental lifetimes scaled to the range [0, 1],

1 $$\begin{aligned} R_\tau (z_{\hbox {F}})=\frac{\langle \tau \rangle (z_{\hbox {F}})-\tau _{\hbox {B}}}{\langle \tau \rangle (0)-\tau _{\hbox {B}}}, \end{aligned}$$

where $\langle \tau \rangle (z_{\hbox {F}})$ is amplitude averaged lifetime at nominal depth $z_{\hbox {F}}$. The observation motivates the model below.

### Extraction of the contribution of the physisorbed species

It has been known long, but perhaps not widely enough- we learned only from experience, that homogeneous thick media degrade spatial filtering in the confocal microscope. Here, fluorescence of the homogeneous solution above the glass contaminates the interesting signal from the interface and, to a lesser extent, *vice versa*. In general, the summed effect of signal from out of focus planes produces a strong background, that could in principle be accounted for by deconvolution of the depth dependent signal with the instrument point spread function (PSF), see for example refs.^33–35^. Such an approach may be tractable when the objective is used close to its design conditions, particularly the thickness of the cover slip and the refractive index (RI) of the sample medium, usually water, so that the PSF is independent of composition and depth of focus. Deviation of the refractive index of our samples with respect to design, and probing considerable depths, cause variable aberrations at the interface that stretch the ideal PSF far beyond the diffraction limit, precluding such an approach here, not to mention mathematical cumbersomeness.

Our aim is nonetheless to investigate the adsorption equilibrium of 4-daspi as a function of the solvent composition. To do so, we introduce a simple phenomenological model using experimentally accessible data to approximately deconvolute the contributions of adsorbed and free-swimming chromophores. The excitation intensity, $I_{\hbox {ex}}(x,y,z|z_{\hbox {F}})$ at any point (*x*, *y*, *z*) in the sample depends on the depth of the nominal focus into the solution, $z_{\hbox {F}}$, measured from the glass, and on aberrations. Then up to a global, instrument-dependent constant, the steady state intensity from a fluorophore with concentration distribution $\rho (x,y,z)$ (molecules/unit volume) is:

2 $$\begin{aligned} I(z_{\hbox {F}}) = \int \int \int dxdydz \quad I_{\hbox {ex}}(x,y,z|z_{\hbox {F}})\rho (x,y,z)\epsilon \Phi D(x,y,z), \end{aligned}$$

where *D*(*x*, *y*, *z*) is the detection efficiency (fraction of photons emitted over $4\pi$ sr from point (*x*, *y*, *z*) that reach the detector), $\epsilon$ is the extinction coefficient at the excitation wavelength and $\Phi$ is the fluorescence quantum yield. Efficiency *D* depends on the instrument, particularly the objective and the pinhole, and on aberrations introduced by mismatch of refractive index with respect to design. Fortunately, for present work neither $I_{ex}$ nor *D* need to be measured. It is also implicit that the optical density is low enough to neglect the influence of absorption on the *z*-profile of $I_{\hbox {ex}}$. We also neglect (after experimental verification) the luminescence of the glass.

The signal from a homogeneous bulk solution of a non-adsorbing chromophore at concentration $\rho$, lying behind the glass interface (at $z_{\hbox {F}}=0$) is thus:

3 $$\begin{aligned} I_{\hbox {H}}(z_{\hbox {F}}) = \rho \varepsilon \Phi \int \int \int dxdydz \quad I_{\hbox {ex}}(x,y,z|z_{\hbox {F}}) D(x,y,z). \end{aligned}$$

The normalised rise of the signal as the focus plunges into the homogeneous medium is,

4 $$\begin{aligned} H(z_{\hbox {F}}) = I_{\hbox {H}}(z_{\hbox {F}})/I_{\hbox {H}}(0), \end{aligned}$$

an RI-dependent, but experimentally accessible quantity. $H(z_{\hbox {F}})$ rises monotonically with depth $z_{\hbox {F}}$ in index-matched solutions (and $1 \le H(z_{\hbox {F}})\le 2$), but exhibits a broad maximum followed by decline, when index mismatch introduces aberrations.

Here, a homogeneous bulk solution of dye (B) at concentration $\rho$ and a layer physi-sorbed on the glass surface (S, surface density $\sigma$) are present together. With obvious notation, the signal at nominal focus $z_{\hbox {F}}$ is:

5 $$\begin{aligned} I(z_{\hbox {F}})=\sigma \varepsilon _{\hbox {S}}\Phi _{\hbox {S}} \int \int dxdy\quad I_{\hbox {ex}}(x,y,0|z_{\hbox {F}})D(x,y,0) + \rho \varepsilon _{\hbox {B}}\Phi _{\hbox {B}} \int \int \int dxdydz \quad I_{\hbox {ex}}(x,y,z|z_{\hbox {F}})D(x,y,z)\quad . \end{aligned}$$

**Figure 3:**
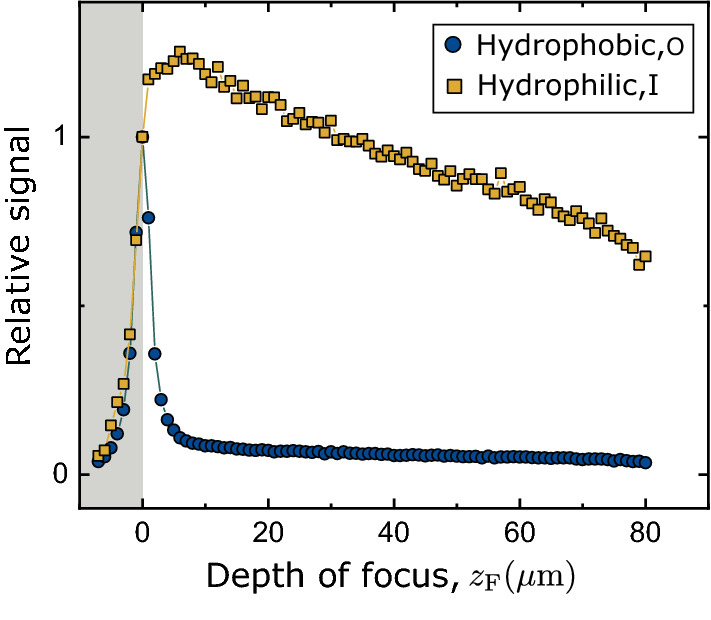
Sample data Relative intensity during a *z*-scan into a 70 wt.% glycerol solution of 4-daspi (white area), from the hydrophobic glass (blue, showing a spike due to the adsorbed dye) or the hydrophilic glass (yellow, no adsorption detected).

In addition to the features of $H(z_{\hbox {F}})$ above, the normalised intensity,

6 $$\begin{aligned} I_{\hbox {N}}(z_{\hbox {F}}) = I(z_{\hbox {F}}) / I(0), \end{aligned}$$

again accessible to experiment, may show a spike at the glass surface due to physisorbed molecules. Figure 3 illustrates this. No adsorption is found on the hydrophilic interface. A spike, coinciding with the increased lifetime with respect to bulk, signals adsorption on the hydrophobic interface.

Comparing $I_{\hbox {N}}(z_{\hbox {F}})$ and $H(z_{\hbox {F}})$, one may therefore derive information on the adsorbed species, specifically the ratio

7 $$\begin{aligned} K = \frac{\sigma \varepsilon _{\hbox {S}}\Phi _{\hbox {S}}}{\rho \varepsilon _{\hbox {B}}\Phi _{\hbox {B}}}. \end{aligned}$$

Ratio *K* and the known properties of the bulk species are used below to extract the surface density of adsorbed molecules, $\sigma$.

Now Eqs. (4)–(6) may be reworked to yield

8 $$\begin{aligned} U(z_{\hbox {F}}) = \frac{I_{\hbox {N}}(z_{\hbox {F}})}{H(z_{\hbox {F}})} = \frac{1 + K\phi (0,z_{\hbox {F}})}{1+K\phi (0,0)} = \frac{\phi (0,0)^{-1}+KR_{\hbox {th}}(z_{\hbox {F}})}{\phi (0,0)^{-1}+K} \quad \le 1, \end{aligned}$$

where

9 $$\begin{aligned} \phi (z,z_{\hbox {F}}) = \frac{\int \int dxdy I_{\hbox {ex}}(x,y,z|z_{\hbox {F}})D(x,y,z)}{\int \int \int dx dy du I_{\hbox {ex}}(x,y,u|z_{\hbox {F}})D(x,y,u) } \end{aligned}$$

and

10 $$\begin{aligned} R_{\hbox {th}}(z_{\hbox {F}}) = \frac{\phi (0,z_{\hbox {F}})}{\phi (0,0)}. \end{aligned}$$

Function $\phi (z,z_{\hbox {F}})$ will be recognised as a probability density, such that the probability that a detected photon arises from anywhere in a slice of a homogeneous sample $[z,z+\delta z]$ is $\phi (z,z_{\hbox {F}})\delta z$ when the nominal focus is at depth $z_{\hbox {F}}$.

The relation between $R_{\hbox {th}}$ and $R_\tau$ is derived in the SI, for the case when fluorescence decays of both the surface and the bulk species are monoexponentials, with lifetimes $\tau _{\hbox {S}}$ and $\tau _{\hbox {B}}$:

11 $$\begin{aligned} \frac{R_{\hbox {th}}(z_{\hbox {F}})}{R_\tau (z_{\hbox {F}})} =\frac{\phi (0,0)^{-1}}{\phi (0,0)^{-1}+\frac{\tau _{\hbox {B}}}{\tau _{\hbox {S}}}K(1-R_\tau (z_{\hbox {F}})) }. \end{aligned}$$

Unfortunately, that is not the case for the fluorescence of 4-daspi in solution, which has biexponential decay, so we revert to Eqs. (7) and (8). As shown in the SI, $\phi (z,z_{\hbox {F}}) \propto 1/|z-z_{\hbox {F}}|^2$ at long range, so we expect *U* to tail off to an asymptote, $U(z_{\hbox {F}})\rightarrow U_\infty =(1+\phi (0,0)K)^{-1}$, from which:

12 $$\begin{aligned} K \approx \frac{1-U_\infty }{\phi (0,0)U_\infty }\quad . \end{aligned}$$

The only item in Eq. (12) that is not accessible to experiment is $\phi (0,0)$, here $\approx 0.2$ $\upmu$m $^{-1}$, which we determine numerically with the help of Monte Carlo ray tracing, using the design parameters of the microscope and accounting for refraction in the sample (SI table S1). It turns out to be a weak function of RI. Although the convergence of U to an asymptote is approximate, the variations of *K* determined this way from *U* at different $z_{\hbox {F}}$ are small compared to the range over which *K* varies between pure water and pure 99% glycerol (see SI Fig. S1).

**Figure 4:**
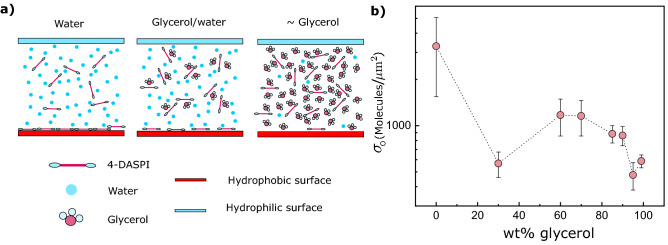
Adsorption of 4-daspi on hydrophobic glass decreases with glycerol concentration (**a**) Schematic representation of how reduction of energetically unfavourable contacts for molecules close to the glass-solution interface may drive the adsorption of 4-daspi to hydrophobic glass. (**b**) Adsorption of 4-daspi to the hydrophobic surface ($\sigma _{\textrm{O}}$) as deduced from confocal intensity *z*-scans, corrected for artefacts as described in the text. Error bars 1 s.d. for data collected at $z_{\hbox {F}}=5,7,10,20,30$ $\upmu$m. The black dashed line is just to guide the eye.

Since the extinction coefficient depends on the ground state conformation, not the TICT state, let us further assume $\varepsilon _{\hbox {S}}\approx \varepsilon _{\hbox {B}}$. Furthermore, in agreement with the literature, let us assume that the viscosity dependences of the fluorescence quantum yield and lifetime are dominated by non-radiative relaxation of the TICT state. Then the convergence of the lifetimes measured here in the bulk and at the hydrophobic interface implies by extrapolation (dashed line in Fig. 1c) a common lifetime and quantum yield at $\eta _{\hbox {I}} \approx 2\times 10^{4}$ mPa.s with quantum yield $\Phi _{\hbox {I}}=0.15$. The fluorescence quantum yield of 4-daspi *vs.* viscosity in glycerol/water solution was reported in ref.^19^. Using those values, we may thus determine $\sigma$ on the hydrophobic surface ($\sigma _{\textrm{O}}$) from the values of K derived *via* Eq. (12), since all remaining quantities in Eq. (7) are known. Fig. 4b shows the dependence of the physio-sorbed surface number density $\sigma _{\textrm{O}}$ on the composition of the solvent.

### Discussion

Physisorption of 4-daspi, from solution in pure water to hydrophilic glass was below the level of detection *via* the fluorescence lifetime measured at the interface, as one might expect from the very ready solubility of the dye, which is an organic salt. However, counter to intuition, physisorption on hydrophobic glass is greatest in pure water at $\sigma _{\textrm{O}}\approx 3300$ $\upmu$m $^{-2}$, or around 0.3% of a monolayer. It is about 10 times smaller in nearly pure glycerol, with a relatively sharp drop between 60 and 100 wt.%. These observations may be rationalised by considering the structure of the dye.

Although 4-daspi is a water-soluble salt, it has a conjugated hydrophobic core. In free solution in water, both faces of the core present ’bad’ contacts, energetically unfavourable hydrophobic-hydrophilic interactions, see Fig. 4a. Little energetic advantage accrues from contact with hydrophilic glass, and there may well be a disadvantage due to displacement of water hydrogen bonded to silanols on the glass. When the same solution is in contact with the silanised, hydrophobic glass, the same movement of the dye replaces two out of three bad contacts- water to dye and water to glass, and introduces a favourable dye-glass interaction. One would therefore expect the dye to be driven to the interface, the final adsorption equilibrium of course being limited by entropic considerations.

In the more concentrated glycerol solutions, the situation is different. Glycerol, with three OH groups balanced by CH$_2$’s or CH, may intermediate between the hydrophobic core of the dye and water. If, plausibly, the 4-daspi core interacts preferentially with the methylene units of glycerol, there would then be no change in the number of bad contacts following adsorption, and the surface density of the dye should diminish with glycerol concentration, as observed.

Physisorption to the hydrophobic interface by van der Waals (dispersion or London) interactions, is expected to reinforce the planarity of the core, preventing formation of the TICT state by internal rotation. Therefore, the fluorescence lifetime increases, as observed.

Interestingly, after reaching a plateau at glycerol concentrations between 30 wt.% and 60 wt.%, the concentration of adsorbed 4-daspi ($\sigma _{\textrm{O}}$) drops off steeply beyond 60 wt.%. The drop may be related to the decrease of acceptor hydrogen bonding in water^36^. In this work, Towey et al. examined glycerol/water hydrogen bonding in glycerol concentrated solutions by molecular dynamics simulation as well as neutron diffraction experiments. Water-water hydrogen bonding is significantly perturbed at high glycerol concentrations, the majority water species being isolated molecules^36–40^. Indeed, the glycerol molecules has the potential to create three hydrogen bonds with water molecules, so that even in an average fashion, all water molecules are surrounded by glycerol at glycerol concentrations over 80 wt.%. Therefore, there is less particular energetic advantage driving 4-daspi to physisorb to the interface. Similarly, nano-scale clustering of species in the mixed solvent might play a role in the adsorption dip at 30  wt.% glycerol^37^. As a final remark, we note that radiative decay is enhanced at the interface with glass^41^, and that the effect is greatest when the contrast in RI is greatest. Part of the apparent excess adsorption in pure water might be related to this effect. However, the increase of radiative decay rate for physio-sorbed on glass under water is only $\approx 10\%$, so stronger adsorption from water and from the glycerol solution remains. A molecular dynamics simulations of the present system might provide a more quantitative insight into the above qualitative considerations.

## Conclusions

In the present work, we used a confocal microscope to exhibit the adsorption of 4-daspi to hydrophobic glass *via* measurement of the longer fluorescence lifetime at the interface than in the bulk the solution. Diminished confocal optical sectioning in this configuration leads to an apparent dye-glass interaction up to 10’s of $\upmu$m from the interface. A simple model, supported by Monte Carlo ray tracing, enables substantial correction of the effect, using essentially experimental data only. The model is applicable to other systems. Here, we applied it to glycerol/water mixtures, to extract the amount of dye adsorbed to the interface. Counter intuitively, adsorption of the cationic, readily water-soluble dye to silanised glass is greatest in pure water, for reasons that could be further explored by molecular simulation.

## Materials and methods

### Materials

Water/glycerol mixtures were prepared by mixing glycerol (99.5%, Alfa Aesar) in ultra-pure water. A large range of concentrations, [0-99] wt.% with the range of viscosity, [1-900] mPa.s were investigated. The stock solution of 4-daspi (from Sigma-Aldrich) is prepared at $10^{-3}$ M in ultrapure water (with a resistance of 18.2 M$\Omega$). The concentration of 4-daspi in water/glycerol solutions is $10^{-7}$ M to avoid dye-dye interactions.

All measurements were done with #1.5H coverslips (Deckgläser, thickness of $170 \pm 5$ $\upmu$m). The hydrophilic surfaces were prepared as follows. Coverslips were washed with ethanol (purity 99%), thoroughly rinsed in ultrapure water, dried in nitrogen and placed in a plasma cleaner for 30s. Hydrophobic surfaces were obtained by soaking the above coverslips for 15 min in a toluene/trichlorooctylsilane mixture (1%vol), rinsing with isopropanol and drying under nitrogen. Prepared glass was used immediately. The viscosity of the glycerol/water solution was measured by Anton-Paar MCR 302 rheometer.

### Confocal microscopy

A droplet of 4-daspi solution was mounted between a pair of coverslips, one hydrophobic, the other hydrophilic, with a $\approx 70 \pm 15 \upmu$m thick spacer. Either interface can be presented to the objective. Time resolved fluorescence decay was measured by time correlated single photon counting (TCSPC) on an inverted confocal microscope (Leica, TCS SP8) with a 20x dry objective (NA = 0.75) and a 56.6 $\upmu$m pinhole. The nominal *z*-resolution is estimated to be $2\,\upmu$m with the used wavelength, pinhole and objective. 4-daspi was excited at a repetition rate 40 MHz, with the instrument’s pulsed 470 nm laser and emission was collected from 500 and 700 nm. The hydrophobic interface was of course investigated by turning the cell over to present it to the objective. The whole thickness of the droplet was scanned in 1 $\upmu$m steps, from the hydrophobic to the hydrophilic surfaces. The TCSPC histograms were all fitted with bi-exponential functions. We report amplitude average lifetimes $\langle \tau \rangle$ defined as:

13 $$\begin{aligned} \langle \tau \rangle = \frac{A_1 \tau _1 +A_2 \tau _2}{A_1 +A_2} \end{aligned}$$

with $A_i$ and $\tau _i$ are the amplitude and lifetime of the i-th decay component, determined by fitting with the instrument software (Supplementary Information).

## Supplementary Information

Supplementary Information.

## Data Availability

The data that support the findings of this study are available from the corresponding author upon reasonable request.

## References

[1] Lakowicz JR (1999). Principles of Fluorescence Spectroscopy.

[2] Valeur B, Berberan-Santos MN (2012). Molecular Fluorescence Principles and Applications.

[3] Zumbusch A, Fleury L, Brown R, Bernard J, Orrit M (1993). Probing individual two-level systems in a polymer by correlation of single molecule fluorescence. Phys. Rev. Lett..

[4] Sesselmann T, Richter W, Haarer D, Morawitz H (1987). Spectroscopic studies of impurity-host interactions in dye-doped polymers: Hydrostatic-pressure effects versus temperature effects. Phys. Rev. B.

[5] Sesselmann T, Kador L, Richter W, Haarer D (1988). Correlation between strain-field and electric-field effects in hole-burning spectra. EPL.

[6] Zhuravlev LT (2000). The surface chemistry of amorphous silica. Zhuravlev model. Colloids Surf. Physicochem. Eng. Asp..

[7] Dong Y, Pappu SV, Xu Z (1998). Detection of local density distribution of isolated silanol groups on planar silica surfaces using nonlinear optical molecular probes. Anal. Chem..

[8] Fan H-F, Li F, Zare RN, Lin K-C (2007). Characterization of two types of silanol groups on fused-silica surfaces using evanescent-wave cavity ring-down spectroscopy. Anal. Chem..

[9] Haidekker MA, Theodorakis EA (2010). Environment-sensitive behavior of fluorescent molecular rotors. J. Biol. Eng..

[10] Loutfy RO, Arnold BA (1982). Effect of viscosity and temperature on torsional relaxation of molecular rotors. J. Phys. Chem..

[11] Kim J, Lee M (1999). Excited-state photophysics and dynamics of a hemicyanine dye in AOT reverse micelles. J. Phys. Chem. A.

[12] Haidekker MA, Brady TP, Lichlyter D, Theodorakis EA (2005). Effects of solvent polarity and solvent viscosity on the fluorescent properties of molecular rotors and related probes. Bioorganic Chem..

[13] Vyšniauskas A (2017). Exploring viscosity, polarity and temperature sensitivity of BODIPY-based molecular rotors. Phys. Chem. Chem. Phys..

[14] Kang J, Lhee S, Lee JK, Zare RN, Nam HG (2020). Restricted intramolecular rotation of fluorescent molecular rotors at the periphery of aqueous microdroplets in oil. Sci. Rep..

[15] Loutfy RO, Teegarden DM (1983). Effect of polymer chain tacticity on the fluorescence of molecular rotors. Macromolecules.

[16] Law KY, Loutfy RO (1983). Fluorescence probe for microenvironments: On the fluorescence properties of p-N, N-dialkylaminobenzylidenemalononitrile in polymer matrices. Polymer.

[17] Vyšniauskas A (2021). Cyclopropyl substituents transform the viscosity-sensitive BODIPY molecular rotor into a temperature sensor. ACS Sens..

[18] Huang Y (2002). Photophysical studies on the mono- and dichromophoric hemicyanine dyes II. Solvent effects and dynamic fluorescence spectra study in chloroform and in LB films. J. Phys. Chem. B.

[19] Shim T, Lee MH, Kim D, Ouchi Y (2008). Comparison of photophysical properties of the hemicyanine dyes in ionic and nonionic solvents. J. Phys. Chem. B.

[20] Pillai ZS, Sudeep PK, Thomas KG (2003). Effect of viscosity on the singlet-excited state dynamics of some hemicyanine dyes. Res. Chem. Intermed..

[21] Görner H, Gruen H (1985). Photophysical properties of quaternary salts of 4-dialkylamino-4-azastilbenes and their quinolinium analogues in solution. J. Photochem..

[22] Jedrzejewska B, Kabatc J, Pietrzak M, Paczkowski J (2003). Hemicyanine dyes: Synthesis, structure and photophysical properties. Dyes Pigments.

[23] Jee A-Y, Bae E, Lee M (2010). Internal motion of an electronically excited molecule in viscoelastic media. J. Chem. Phys..

[24] Bittermann MR, Grzelka M, Woutersen S, Brouwer AM, Bonn D (2021). Disentangling nano- and macroscopic viscosities of aqueous polymer solutions using a fluorescent molecular rotor. J. Phys. Chem. Lett..

[25] Briole A, Podgorski T, Abou B (2021). Molecular rotors as intracellular probes of red blood cell stiffness. Soft Matter..

[26] Wirth MJ, Swinton DJ, Ludes MD (2003). Adsorption and diffusion of single molecules at chromatographic interfaces. J. Phys. Chem. B.

[27] Li Z, Ashraf KM, Collinson MM, Higgins DA (2017). Single molecule catch and release: potential-dependent plasmid DNA adsorption along chemically graded electrode surfaces. Langmuir.

[28] Wang D, Schwartz DK (2020). Non-brownian interfacial diffusion: flying, hopping, and crawling. J. Phys. Chem. C.

[29] Seebacher, C. F. *Einzelmolekülspektroskopie von organischen Farbstoffmolekülen in porösen Festkörpern und Tieftemperaturspektroskopie an dem grün fluoreszierenden Protein*. Ph.D. thesis, Ludwig-Maximilians-Universität München (2002).

[30] Förster T, Hoffmann G (1971). Molecular rotors. Z. Phys. Chem.

[31] Pandey SP (2021). A cyanine based dicationic molecular rotor probe for dual sensing of heparin. J. Mol. Liq..

[32] Singh G (2021). Sulfated-β-cyclodextrin templated aggregation of a metachromatic dye, Basic Orange 21: A photophysical investigation. Supramol. Chem..

[33] Stevens JK, Mills LR, Trogadis JE (1994). Cell Biology. Three-dimensional Confocal Microscopy.

[34] Gu M (1996). Principles of Three Dimensional Imaging in Confocal Microscopes.

[35] Kirshner H, Aguet F, Sage D, Unser M (2013). 3-D PSF fitting for fluorescence microscopy: Implementation and localization application. J. Microsc..

[36] Towey JJ, Soper AK, Dougan L (2011). Preference for isolated water molecules in a concentrated glycerol–water mixture. J. Phys. Chem. B.

[37] Towey JJ, Soper AK, Dougan L (2013). What happens to the structure of water in cryoprotectant solutions?. Faraday Discus..

[38] Towey JJ, Soper AK, Dougan L (2016). Low-density water structure observed in a nanosegregated cryoprotectant solution at low temperatures from 285 to 238 K. J. Phys. Chem. B.

[39] Towey J, Soper A, Dougan L (2012). Molecular insight into the hydrogen bonding and micro-segregation of a cryoprotectant molecule. J. Phys. Chem. B.

[40] Wu K, Feng S, Hedoux A, Shalaev E (2022). Water structure in glycerol: Spectroscopic and computer simulation investigation of hydrogen bonding and water clustering. J. Mol. Liq..

[41] Lukosz W, Kunz RE (1977). Light emission by magnetic and electric dipoles close to a plane interface. I. Total radiated power. J. Opt. Soc. Am. JOSA.

