# Supplementary material for: Adsorption of a water-soluble molecular rotor fluorescent probe on hydrophobic surfaces
Source: Sci Rep. 2022 Dec 23;12:22197. doi: 10.1038/s41598-022-26722-w (PMC9789158; doi:10.1038/s41598-022-26722-w)
Supplement: Supplementary file 1 — Supplementary Information. [file 41598_2022_26722_MOESM1_ESM.pdf]

SUPPLEMENTARY INFORMATION
Adsorption of a water-soluble molecular rotor fluorescent probe on hydrophobic surfaces

Elham Mirzahossein[1,*], Marion Grzelka[1], Fabrice Guerton[2], Daniel Bonn[1], and Ross Brown[3]

[*]e.mirzahossein@uva.nl
[1]Van der Waals-Zeeman Institute, Institute of Physics, University of Amsterdam, 1098XH Amsterdam, The Netherlands
[2]Université de Pau et des Pays de l'Adour, E2S UPPA, CNRS, IPRA, Pau, France
[3]Université de Pau et des Pays de l'Adour, E2S UPPA, CNRS, IPREM, Pau, France

1 Form of the function $\phi(z, z_F)$

Confocal sectioning

Aberrations and ray optics dominate diffraction limited propagation in our non-RI-matched samples. Therefore, in the model, we replace the actual Gaussian beam by a classical beam that would have the same numerical aperture and waist in the objective front focal plane in an index-matched sample. Then we apply ray optics and ultimately Monte Carlo simulation to model the nearing and stretching of the focus by refraction in non-RI matched media. Filling the back focal plane with Köhler illumination with very small vergence produces the required classical waist, which is bi-conical rather than Gaussian.

Consider first an index-matched medium, *i.e.* for which the objective was designed to function as a simple thin lens. Figure S1a schematically shows a confocal fluorescence microscope with objective and tube focal lengths f_{obj} and f_{tube}, providing lateral and axial magnifications $M = f_{\text{tube}}/f_{\text{obj}}$ and M^2. Let P be the power through the objective, focused with numerical aperture NA$= \sin\alpha$ to a waist diameter w, figure S1b). Introducing the intensity at the waist, $I_w = 4P/(\pi w^2)$, the intensity at any point P out of focus by an algebraic amount z and a distance r off the axis is given by

$$I_{\text{ex}}(z) = I_w \left(\frac{w}{w+2|z|\tan\alpha}\right)^2 \quad , r \leq w/2 + |z|\tan\alpha \quad , \tag{1}$$

$$= 0 \quad \text{otherwise.} \tag{2}$$

Figure S1c shows the vicinity of the pinhole at the conjugate focus to the excitation beam waist. As usual, we assume the pinhole diameter, h, is close to that of the diffraction limited image spot, $h = w' = Mw$. We again approximate the diffraction limited image of a point P(z,r) by a classical waist w' lying at P'(z',r'), where $r' = Mr$, but the defocusing relative to the pinhole is $z' = M^2 z$. Thus the intensity collected from a point emitter at P is spread over a disk in the pinhole plane, radius $R' = w'/2 + z'\tan\alpha' = M(w/2 + z\tan\alpha)$ (figure S1c). The detection efficiency, $D(z,r)$, for fluorescence from point $P(z,r)$ is proportional to the fraction of the disk overlapping the pinhole, so (neglecting partial overlap for points at the edge of the illuminated field):

$$D(z,r) = D(z) = \left(\frac{w'}{2R'}\right)^2 = \frac{w^2}{(w+2|z|\tan\alpha)^2} \quad , r \leq w/2 + |z|\tan\alpha \quad . \tag{3}$$

The signal corresponding to a fluorophore with number density $N(x,y,z)$, extinction coefficient ε and fluorescence quantum yields Φ is thus proportional to

$$S = \int\int\int_{\text{illuminated volume}} dxdydz I_{\text{ex}}(z) \times \varepsilon\Phi N(x,y,z) \times D(z) \quad , \tag{4}$$

$$= I_w \varepsilon\Phi \int_{-\infty}^{\infty} dz \frac{w^2}{(w+2|z|\tan\alpha)^2} \frac{w^2}{(w+2|z|\tan\alpha)^2} \int\int_{\mathscr{D}(z)} dxdy N(x,y,z) \quad , \tag{5}$$

where $\mathscr{D}(z)$ is the illuminated disk at defocusing z, diameter $w + 2|z|\tan\alpha$.

Three cases are relevant here:

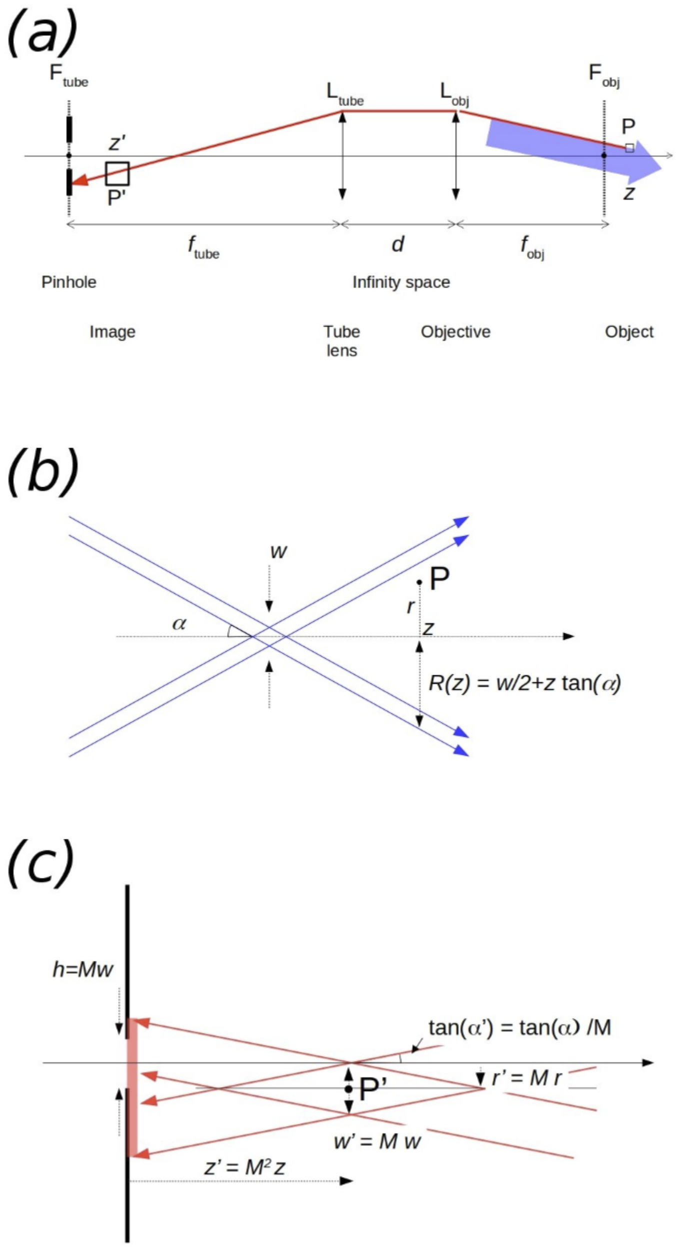

Figure S1. **Spatial filtering in the confocal fluorescence microscope** *(a)* A point P illuminated with blue light and a ray of red fluorescence contributing to its image at point P'; *(b)* The vicinity of the focus; *(c)* The vicinity of the pinhole.

- *Point fluorophore* For a point emitter such as a single molecule in a non-fluorescent medium, lying on the optical axis at defocusing Δz, $N(x,y,z) = \delta(x)\delta(y)\delta(z-\Delta z)$ and eq. (5) reduces to :

$$\frac{S_{1\,\mathrm{mol}}(\Delta z)}{I_w \varepsilon \Phi} = \left(\frac{w}{w + 2|\Delta z|\tan\alpha}\right)^4 \quad . \tag{6}$$

  The severe attenuation of signal from out of focus fluorophores, $\propto 1/\Delta z^4$, is the origin of spatial filtering in confocal microscopes.

- *Layer of fluorophores* For a uniform 2-d distribution of fluorophores at defocusing Δz, *e.g.* those adsorbed here on the glass, with surface density σ, we have $N(x,y,z) = \sigma\delta(z-\Delta z)$ and:

$$\frac{S_{\mathrm{layer}}(\Delta z, \sigma)}{I_w \varepsilon \Phi} = \frac{\pi w^2}{4}\sigma\left(\frac{w}{w + 2|\Delta z|\tan\alpha}\right)^2 \quad , \tag{7}$$

  which decays slower, as $1/\Delta z^2$: spatial filtering is still present, but much less effective.

- *Fluorescent slab* For a semi-infinite fluorescent slab, *e.g.* the present bulk solutions, with interface lying at $z = \Delta z$, $N(x,y,z) = \rho\Theta(z-\Delta z)$, where Θ is the Heavyside step function and ρ is the bulk number concentration. Then

$$\frac{S_{\mathrm{slab}}(\Delta z, \rho)}{I_w \varepsilon \Phi} = \frac{\pi w^3 \rho}{8\tan\alpha} \times \frac{w}{w + 2\Delta z \tan\alpha} \quad , \Delta z \geq 0 \quad \text{(Slab ahead of the waist, \textit{i.e.} focus outside sample)}, \tag{8}$$

$$= \frac{2S_{\mathrm{slab}}(0) - S_{\mathrm{slab}}(-\Delta z)}{I_w \varepsilon \Phi} \quad , z < 0 \quad \text{(Focus inside sample)}, \tag{9}$$

  where the second relation follows from geometrical arguments. Neglecting the effects of refraction, the signal thus rises slowly, $\propto 1/\Delta z$, as the focus approaches the interface, where it reaches half the saturation value attained asymptotically as the focus plunges deep into the medium. In practice aberrations due to RI mismatch at the interface commonly cause the signal to rise to a broad maximum just inside the interface, followed by a slow decay at greater depth.

In all these expressions, the selectivity increases with the numerical aperture, increasing angle α.

We now ask when a layer of adsorbed molecules (ads) might be detected against the bulk background intensity as the focus is swept across the glass-solution interface at $\Delta z = 0$, by setting:

$$\frac{S_{\mathrm{layer}}}{S_{\mathrm{slab}}} = \frac{2\tan(\alpha)\sigma\varepsilon_{\mathrm{ads}}\Phi_{\mathrm{ads}}}{w\rho\varepsilon_{\mathrm{slab}}\Phi_{\mathrm{slab}}} > 1 \quad . \tag{10}$$

Setting an area ≈ 1 nm^2 for a typical dye, $w \approx 0.5\ \mu$m and expressing σ in monolayers and ρ in molar concentration,

$$\sigma > 300\rho\frac{\varepsilon_{\mathrm{slab}}\Phi_{\mathrm{slab}}}{\varepsilon_{\mathrm{ads}}\Phi_{\mathrm{ads}}} \quad , \tag{11}$$

from which one would expect a detection threshold of a few 10^{-3} of a mono-layer.

These considerations are illustrated by experimental data in Fig.4b of the main text.

Form of function $\phi(z, z_F)$

The above formulae now provide estimates of function $\phi(z, z_{\mathrm{F}})$. Considering a homogeneous slab sample, and associating a "surface density" σ with an infinitesimal slice $[z, z+dz]$, such that $\sigma = \rho dz$, the probability distribution of detected photons arising from depth z is given by eqs. (7, 9):

$$\phi(z, z_{\mathrm{F}})dz = \frac{S_{\mathrm{layer}}(|z - z_{\mathrm{F}}|, \rho dz)}{S_{\mathrm{slab}}(z_{\mathrm{F}}, \rho)} \quad . \tag{12}$$

Considering the rise of S_{slab} to a constant when the focus is inside the slab, and the form of 7, one expects $\phi(z, z_{\mathrm{F}}) \propto 1/|z - z_{\mathrm{F}}|^2$ at long range.

In particular:

$$\phi(0, z_{\mathrm{F}}) = \frac{2\tan(\alpha)w}{(w + 2z_{\mathrm{F}}\tan\alpha)(w + 4z_{\mathrm{F}}\tan\alpha)} \tag{13}$$

$$\phi(0,0) = 2\tan(\alpha)/w \quad . \tag{14}$$

Model objective (doublet, separation 40.)		
Front lens	Diam. 1.078	Focal length 1.012
Rear lens	Diam. 15.	Focal length 43.38
Equivalent focal length	10.0	
Working distance	0.78	
N.A.	0.75	
Infinity space	200.	
Perfect long pass filter	Diam. 20.0	
Tube lens	Diam. 25.0	Focal length 200.0
Pinhole	Diam. 0.0565	
Detector	Diam. 1	

Table S1. Microscope model Prescription for modelling experiments with the LEICA TCS SP8 microscope, using the HC PL APO 20x NA 0.75 CS2 dry objective, designed for aqueous media behind #1.5 coverslips. All dimensions in mm.

2 Monte Carlo ray tracing for $\phi(z, z_F)$

We use our in-house ray tracing software MOCARTSI[1] to simulate the experiments. MOCARTSI uses Monte Carlo sampling to launch rays into the system and to determine matter-ray interactions. Straight line propagation is assumed (*i.e.* homogeneous media), except at interfaces, where rays are refracted or reflected according to Snell-Descarte's laws and the polarisation-dependent Fresnel relations. Simulation of ray propagation proceeds by a sequence of events, mostly occurring at interfaces. Rays are traced indefinitely, in any direction, until they are absorbed, hit a detector or escape to infinity. A real microscope objective may contain 10 or more pieces of glass with various curvatures and refractive indices, but it is modelled here by a doublet in the paraxial approximation, yielding the same external parameters: working distance, effective focal length and numerical aperture. That is at first sight paradoxical, but readers will recall that real microscopes are designed to conform as far as possible to that approximation, at least when used close to design conditions. The diffraction limited beam waist at the front focal plane of a real microscope is simulated here by filling the back focal plane of a two-lens model of the objective (see the SI) with a model Köhler illumination with very small numerical aperture ($\approx 10^{-4}$), producing a waist a few hundred nm across.

Additionally, ray-matter interactions may occur at some point along a ray, before the next interface. Absorption is simulated by drawing propagation lengths in a medium from an exponential distribution of random numbers, such that on average average simulated rays obey the Beer-Lambert-Bouguer law. A fraction of absorption events determined by the quantum yield give rise to new rays simulating fluorescence, which is isotropic and unpolarized in the current version of the program, clearly an approximation in the most viscous solutions. An option to log all detected rays, here those falling on a detector behind the pinhole, enables evaluation of $\phi(z, z_\mathrm{F})$ required here.

Here we simulate experiments with the HC PL APO 20x NA 0.75 CS2 objective. Tables S1 and S2 provide the parameters.

3 Computing the adsorbed density, σ

For computing σ, we can use $\sigma = \frac{K\rho\Phi_\mathrm{B}}{\Phi_\mathrm{S}}$. ρ is the concentration of 4-Daspi inside solutions. Φ_B and Φ_S can be estimated by using the measured fluorescence quantum yield of 4-DASPI inside glycerol solution with the viscosity of 900 mPa.s with the value of 0.077[2]. By fitting the FH equation to lifetime as a function of viscosity in bulk ($\tau_{bulk} = 0.23\eta^{0.22}$) for the whole range (1-900 mPa.s) and using the fact that $\phi \sim \tau \sim \eta^b$ we can estimate quantum yield of different viscosity. Moreover we can fit the lifetime on hydrophobic surface as a function of viscosity($\tau_\mathrm{surface} = 1.12\eta^{0.06}$). By extrapolating the the fitting of FH on surface and in bulk we can estimate the quantum yield of intersect point ($\Phi_\mathrm{intersect} = 0.15$ and $\eta_\mathrm{intersect} = 2\times10^4$ mPa.s) and compute the quantum yield of the daspi on hydrophobic surface. K can be calculated by Eq.12 in the main text. At it is shown in Fig.2, $U(z_F)$ converge to an approximate constant value ($U(z_F) \to U_\infty$) for each glycerol/water concentration.

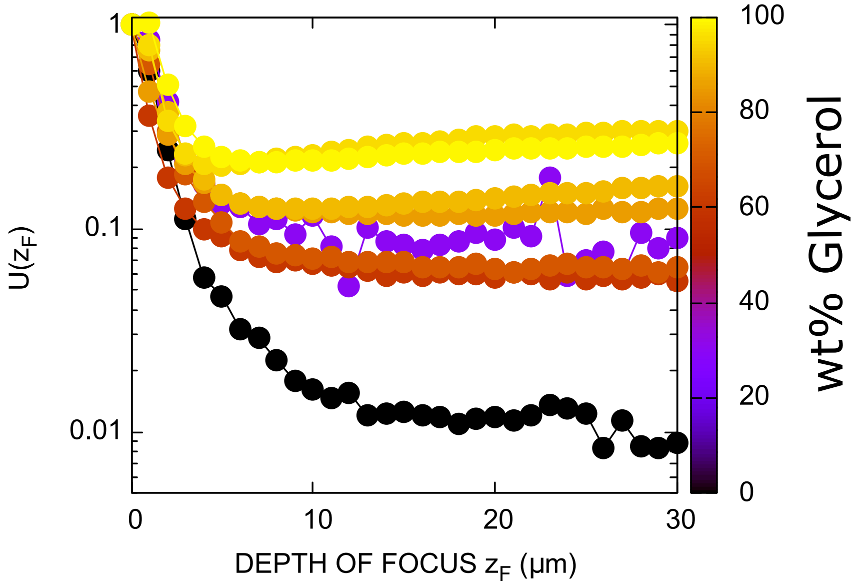

Figure S2. Axial intensity profile with *vs.* without adsorption Function U, eq.8 of the main text captures information on the adsorbed species by comparing the experimental axial fall-off of the fluorescence signals with and without adsorption. The steeper is the fall-off, the higher the adsorbed concentration.

Material	RI
Air	1.0000
water	1.3303
glycerol 100%	1.47399
95%	1.46597
etc.	taken from ref.[3]
BK7 glass	1.5236

Table S2. Refractive indices, RI, of the materials in the simulations

4 Relation between R_{th} and R_τ, eq. 11 of the main text, for mono-exponential decays

Using the notation of the main text, one may write the response of the sample at delay t after an impulse excitation (nominal focus z_F) as sum of surface and bulk contributions. Each is proportional to the initial populations of excited molecules, proportional to $\sigma\varepsilon_S$ and $\rho\varepsilon_B$, their exponentially decaying survival probabilities at time t and to the radiative decay rates, so:

$$\begin{aligned} i(t|z_F) &= i_S(t|z_F) + i_B(t|z_F) && (15)\\ &= A_S(z_F)\exp(-t/\tau_S) + A_B(z_F)\exp(-t/\tau_B) \quad , && (16)\end{aligned}$$

where

$$\begin{aligned} A_S(z_F) &= \sigma\varepsilon_S k_S^r \int\int dxdy \quad I_{ex}(x,y,0|z_F)D(x,y,0) \quad , && (17)\\ A_B(z_F) &= \sigma\varepsilon_B k_B^r \int\int\int dxdydz \quad I_{ex}(x,y,z|z_F)D(x,y,z) && (18)\end{aligned}$$

and k_i^r is the radiative decay rate of species i.

The amplitude averaged lifetime is thus

$$<\tau>(z_F) = \frac{A_S(z_F)\tau_S + A_B(z_F)\tau_B}{A_S(z_F) + A_B(z_F)} \quad . \tag{19}$$

Then R_τ may be expressed as

$$R_\tau(z_F) = \frac{<\tau>(z_F) - \tau_B}{<\tau>(0) - \tau_B} = \frac{A_S(z_F)}{A_S(z_F) + A_B(z_F)} \Big/ \frac{A_S(0)}{A_S(0) + A_B(0)} \quad . \tag{20}$$

Using the definitions (17-18), one finds

$$R_\tau(z_F) = \frac{\sigma\varepsilon_S k_S^r \phi(0,z_F)}{\sigma\varepsilon_S k_S^r \phi(0,z_F) + \rho\varepsilon_B k_B^r} \quad . \tag{21}$$

Introducing the relations between the lifetime,τ, the quantum yield, Φ, and the radiative and non-radiative decay constants, k^r, k^{nr}:

$$\begin{aligned} 1/\tau &= k^r + k^{nr} \quad , && (22)\\ \Phi &= k^r/(k^r + k^{nr}) \quad , && (23)\end{aligned}$$

eq. 11 of the main text readily follows:

$$\frac{R_{\text{th}}(z_F)}{R_\tau(z_F)} = \frac{\phi(0,0)^{-1}}{\phi(0,0)^{-1} + \frac{\tau_B}{\tau_S}K(1 - R_\tau(z_F))} \quad . \tag{24}$$

5 Effect of polarity on fluorescence lifetime of 4-DASPI

Both polarity and viscosity are changing with the glycerol content and might affect the lifetime measurements. To disentangle these effect, we measured fluorescence lifetimes of 4-DASPI in ethanol/water mixtures for which the dielectric constant change in the same range as the water/glycerol mixture but the viscosity is rather constant. Indeed, the dielectric constant of pure water is 78.36, glycerol is 42.5 at T= 298.15 K and a mixture of water/glycerol varies between these two values[4]. As it is shown in the figure S3, there is no apparent change in fluorescence lifetime in this range of dielectric constant. Therefore the dominant factor that affects the molecular dynamics of fluorescence dye in our measurements is viscosity. These results are consistent with fluorescence intensity measurements presented in supplementary materials in Bittermann *et al.*[5].

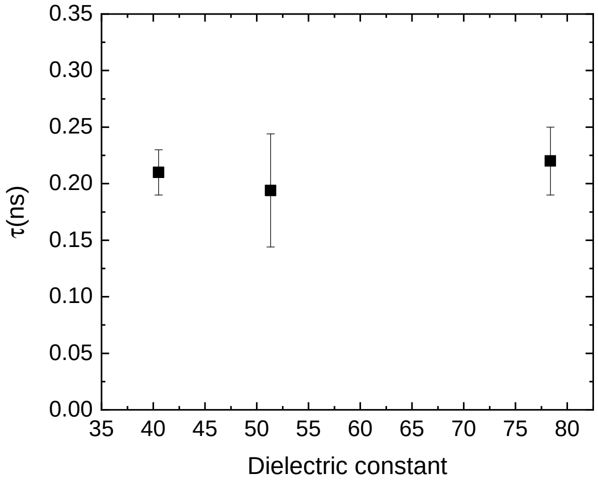

Figure S3. Fluorescence lifetime of 4-DASPI inside ethanol/water mixture as a function of dielectric constant.

References

1. Guerton, F. & Brown, R. Mocartsi: a general Monte Carlo ray tracing program. *To be released under CeCILL B public licence* (2022).
2. Shim, T., Lee, M. H., Kim, D. & Ouchi, Y. Comparison of Photophysical Properties of the Hemicyanine Dyes in Ionic and Nonionic Solvents. *J. Phys. Chem. B* **112**, 1906–1912, DOI: 10.1021/jp076757v (2008).
3. Refractive Index of Glycerine-Water Solutions at 20°C. Http://edge.rit.edu/edge/P13051/public/Research%20Notes/refractive%20index%20
4. Solubility enhancement of cox-ii inhibitors by cosolvency approach. Babu, PR Sathesh, et al. "Solubility enhancement of cox-II inhibitors by cosolvency approach." Dhaka University Journal of Pharmaceutical Sciences 7.2 (2008): 119-126.
5. Bittermann, M. R., Grzelka, M., Woutersen, S., Brouwer, A. M. & Bonn, D. Disentangling Nano- and Macroscopic Viscosities of Aqueous Polymer Solutions Using a Fluorescent Molecular Rotor. *J. Phys. Chem. Lett.* **12**, 3182–3186, DOI: 10.1021/acs.jpclett.1c00512 (2021).
